# No equal opportunity for leukemia initiating cells

**DOI:** 10.18632/oncotarget.26454

**Published:** 2018-12-14

**Authors:** Tom Verbiest, Simon Bouffler, Christophe Badie

**Affiliations:** Cancer Mechanisms and Biomarkers Group, Radiation Effects Department, Centre for Radiation, Chemical and Environmental Hazards, Public Health England, Didcot, United Kingdom

**Keywords:** leukemia, hematopoietic stem cells, radiation, leukemogenesis, gender

A clear association between radiation exposure and increased incidence of leukemia has been widely described [1]. Over the past decades, various investigations into the changes taking place in the haematopoietic system following initial radiation exposure to physical presentation of leukemia have been completed. Previously, mice had to be sacrificed for the studies and bone marrow cells fixed after which chromosomal aberrations such as interstitial chromosome 2 (Chr2) deletions and *Sfpi1* (PU.1) loss could be assessed by FISH [2, 3] or CGH [4], resulting in a substantial use of mice and a loss of valuable information as it did not allow longitudinal tracking of Chr2 deleted cells over time. Very recently, it has been reported that, using a specifically engineered CBA *Sfpi1*^mCh/GFP^ mouse model carrying a mCherry or GFP fluorescent marker located in the Chr2 minimal deleted region, interstitial Chr2 deletions could be detected for the first time in haematopoietic cells *in vivo* by longitudinal blood sampling. This allowed for tracking of Chr2-deleted cells over time and a continuous assessment of the changes following Chr2 deletions and the progression to overt radiation-induced acute myeloid leukemia (rAML) [5].

Monthly blood sampling of whole body irradiated male CBA *Sfpi1*^mCh/GFP^ mice provided novel insights into leukemia progression and presentation following radiation exposure. Firstly, focusing on mCherry fluorescence loss in individual cells, it was observed that the percentage of mice with mCherry- clonal expansion in peripheral blood white blood cells increased with time and reached 100% at 21 months following radiation exposure. Interestingly, the phenotype of mCherry- clonal expansion changed over time: whereas at 12 months mice presented with mixed lineage mCherry- clonal expansion only, the percentage of mice with lymphoid mCherry- clonal expansion increased over time. Increased GFP expression following radiation-induced deletion of the Rosa26-mCherry construct was detected implying that *Sfpi1* expression is autoregulated, and is in line with previous reports that both the proximal promotor of *Sfpi1* and the URE have binding sites for *Sfpi1* itself [6]. Only when *Sfpi1* expression was no longer upregulated (i.e. detected as identical GFP levels in mCherry- cells compared to mCherry+ cells), changes in white blood cell size and granularity could be detected in the peripheral blood. These changes could always be detected about three weeks before the mouse presented with outward physical signs of AML. All but one male leukemia cases were categorized as myeloid leukemia with an average latency of 11 months between radiation exposure and rAML presentation (9% of male mice). The myeloid leukemias observed in this study were consistent with the two-hit model previously postulated [7]: firstly an interstitial chromosome 2 deletion occurs accompanied by loss of *Sfpi1* and the Rosa26-mCherry or GFP construct, followed by a point mutation in the remaining *Sfpi1* copy.

**Figure 1:**
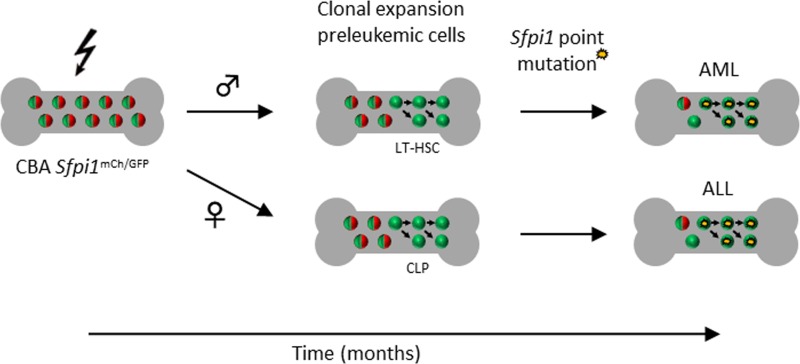
Leukemic pathways observed in CBA *Sfpi1*^mCh/GFP^ mice following radiation exposure LT-HSC, long-term haematopoietic stem cell; CLP, common lymphoid progenitor; AML, acute myeloid leukaemia; ALL, acute lymphoid leukemia.

The vast majority of leukemia induction experiments have been performed using male mice only. The very limited number of studies which did include female mice, consistently reported a lower rAML incidence in female mice. However, an increased leukemia incidence for both men and women is reported in the atomic bomb survivors. Therefore, the study similarly assessed the changes taking place in the haematopoietic system between radiation exposure of female CBA *Sfpi1*^mCh/GFP^ mice and the physical presentation with leukemia. Female mice with lymphoid mCherry- clonal expansion were identified as early as 9 months following radiation exposure, and from 15 months, the percentage of female mice with lymphoid mCherry-clonal expansion was greater than the percentage of female mice with mixed lineage mCherry- clonal expansion. In line with the above-mentioned studies, only one female mouse was diagnosed with rAML. This first observation that the majority of female mice have lymphoid, rather than mixed lineage mCherry- clonal expansion provides at least a partial explanation for the lower rAML presentation observed in female mice compared with male mice. However, seven female mice developed leukemia with a lymphoid phenotype (Figure 1). A potential explanation lies within the observation that in mice with mixed lineage mCherry- clonal expansion, mCherry- haematopoietic stem cells (HSCs) were consistently observed. However, in mice with lymphoid mCherry- clonal expansion, although all HSCs had retained mCherry expression, mCherry- common lymphoid progenitors could be identified. It is possible that a gender-specific radiation-induced chronic expression of pro-inflammatory cytokines could be involved, as it was previously reported that interleukin-1 drives HSCs towards myeloid differentiation at the expense of self-renewal [8].

In conclusion, this first description of a mouse model of radiation leukemia where it is possible to track leukemogenic events over time from initial exposure to presentation of disease provides a basis from which to study additional factors modulating radiation leukemia incidence. For example, how predisposition in human or mouse, exposure to pathogens, inflammation or calorie restriction can modify mouse radiation leukemia incidence [9, 10]. Are the Chr2 deletion and subsequent mutation events directly related to target cell-intrinsic events, or could microenvironment-derived diffusible factors or microvesicle mediation play a role? As pathways of leukemogenesis in mouse and human share substantial commonality, can better approaches to developing counter-measures that reduce cancer incidence following radiation exposure be developed through targeting of specific events associated with leukemogenesis? Furthermore, transplantation studies introducing irradiated or non-irradiated CBA *Sfpi1*^mCh/GFP^ HSCs into hosts exposed or non-exposed could help to further elucidate the relative importance of micro-environmental influences by contrast with cell-intrinsic processes. Each of the above issues could have relevance for radiological protection or help identify cancer-mitigating strategies following radiotherapy, where the development of secondary AML is a clinical problem, or other accidental radiation exposures. Therefore, this publication describes an important model which paves the way for further elucidation of fundamental and applied issues in induced leukemogenesis.
